# Quality of Initial MRI Reports for Soft-Tissue Sarcomas and Their Association with Radiologic Recognition of Sarcoma

**DOI:** 10.3390/diagnostics16193130

**Published:** 2026-09-26

**Authors:** Eric Kholodovsky, Jacob Jahn, Aneesh Samineni, Sergio J. Torralbas Fitz, Jean Jose, Ty Subhawong, H. Thomas Temple

**Affiliations:** 1 Department of Orthopedics, University of Miami, 1120 NW 14th Street, Suite 1263, Miami, FL 33136, USA; 2 Miller School of Medicine, University of Miami, Miami, FL 33136, USA; 3 Department of Radiology, University of Miami, Miami, FL 33136, USA

**Keywords:** MRI, soft-tissue sarcoma, radiologist, score report

## Abstract

**Objectives**: Although MRI is the preferred modality for evaluating suspected soft-tissue sarcomas (STSs), initial MRI reports are frequently incomplete and often fail to document key features that support diagnosis, staging, and timely referral. This study assessed the quality of initial MRI reports for STSs and identified the specific reporting characteristics most strongly associated with diagnostic correlation. **Methods**: A retrospective review was performed of 100 patients with histologically confirmed STSs presenting to a specialized sarcoma center between 2021 and 2024. Initial MRI reports performed externally were analyzed for 19 characteristics based on the European Society of Musculoskeletal Radiology guidelines. Reports were stratified by radiologist fellowship training. Findings were compared across groups, and individual MRI features were assessed for correlation with final histopathologic diagnosis. **Results**: Overall, initial MRI reporting was incomplete, with most features documented in fewer than 70% of cases. Reports by MSK radiologists were higher in quality, more often included bone involvement, peritumoral edema, and suspicion of malignancy. Diagnostic correlation was significantly greater for MSK radiologists compared to general radiologists (*p* = 0.027). Documentation of heterogeneity, depth, gadolinium use, necrosis, and suspicion of malignancy were all strongly associated with diagnostic correlation. **Conclusions**: Initial MRI reports for STSs frequently lack key diagnostic features, but MSK fellowship–trained radiologists produce higher-quality reports and demonstrate stronger diagnostic concordance. Standardized reporting frameworks may ultimately improve patient care and would benefit from direct evaluation in future studies.

## 1. Introduction

Soft-tissue sarcomas (STSs) are a heterogeneous group of more than 50 histologically unique tumors that arise from many different tissue types, including adipose, cartilage, skeletal muscle, smooth muscle, vascular, nerve sheath, synovium, and more [1]. They comprise the majority of primary sarcomas, with a 4:1 ratio compared to bone sarcomas, and have an incidence rate of 7.1 cases per 100,000 people [2]. STSs are aggressive tumors, given their estimated all-cause mortality rate of nearly 34.7% [2]. Early and accurate diagnosis is crucial for improved outcomes in STSs; therefore, strong diagnostic modalities are essential [3,4].

Clinical presentation alone is insufficient for diagnosis and characterization of STSs due to heterogeneity in presentation; therefore, proper imaging techniques and biopsy are required to aid in diagnosis and guide treatment [1,4]. Magnetic resonance imaging (MRI) is currently one of the most valuable imaging modalities for diagnosing STSs, owing to its excellent soft-tissue contrast and detailed anatomical resolution [5,6]. Features such as a deep-seated, large mass, heterogeneous signal characteristics, surrounding edema, and bone involvement can indicate a possible STS. MRI is essential for staging, and as a result, biopsy and surgery are rarely performed before one is obtained [6,7]. Additionally, MRI delineates the relationship of the tumor to adjacent anatomical structures, providing critical information for assessing surgical resectability [6,8].

MRI interpretations often omit clinically relevant features that are essential for accurate diagnosis. A quality assessment study of initial reports found that MRI interpretations frequently deviated from the European Society of Musculoskeletal Radiology guidelines and standard protocol [9]. A dedicated one-year post-residency musculoskeletal radiology fellowship in which radiologists dedicate the entire year to learning to interpret musculoskeletal imaging may help radiologists with STS identification and improving report quality; however, this has not been directly studied, and there is significant heterogeneity in training curriculum and quality among programs [10]. Without a thorough and detailed initial MRI report that highlights key features of a malignancy, STSs may go underdiagnosed or misdiagnosed for something more benign [11]. Because initial imaging is often ordered by clinicians outside of orthopedic oncology, the MRI report commonly serves as the key trigger for timely and appropriate referral. A deficient or inaccurate MRI report can lead to inappropriate treatment, including unplanned excisions, positive tumor margins at resection, and failure to refer patients to a specialized sarcoma center—factors that have all been associated with delayed specialty care and worse outcomes [12,13,14]. Beyond conventional anatomic sequences, advanced and quantitative MRI techniques are increasingly used to characterize soft-tissue masses. Diffusion-weighted imaging (DWI) with apparent diffusion coefficient mapping, dynamic contrast-enhanced (DCE) perfusion imaging, MR spectroscopy, and radiomic analyses provide functional information on cellularity and vascularity that complements morphologic assessment and can improve differentiation of benign from malignant lesions in addition to conventional MRIs [6,15].

The existing literature does not sufficiently highlight the critical role of accurate MRI interpretation in the evaluation of patients with suspected soft-tissue sarcomas. Across the radiology and orthopedic oncology literature, several core elements consistently define a high-quality MRI interpretation when soft-tissue sarcoma (STS) is suspected. These elements reflect both diagnostic needs and the information required for staging, biopsy planning, and surgical decision-making. The purpose of our study is to assess the quality of specific key features of initial MRI reports in STS readings and provide a consensus on the key elements of an MRI report when suspecting a soft-tissue mass.

## 2. Methods

This is a retrospective study of 100 patients treated for soft-tissue sarcomas presenting to our specialized sarcoma center from 2021 to 2024. Patients with a definitive histopathological diagnosis of a malignant soft-tissue sarcoma were identified and included consecutively in reverse chronological order of most recent encounter with an orthopedic oncologist. The initial MRI that was performed and read at an outside facility by an attending radiologist was queried for each patient. Patients were excluded if incomplete or no initial MRI report could be retrieved, a tumor was benign, histopathological results were inconclusive, or if a patient was diagnosed with a primary bone sarcoma. This study was approved by our local ethics board: IRB number 20230560, approved on 7 October 2023.

Demographic data collected included date of most recent encounter with an orthopedic oncologist, histopathological diagnosis of the mass, size of the mass, location of the mass, whether the tumor was completely imaged, date of initial MRI report, type of referring provider for the initial MRI, and type of radiologist who performed the reading of the initial MRI. Type of radiologist was categorized as either general radiologist or fellowship trained musculoskeletal (MSK) radiologist. MSK radiologists were identified by either publicly available information or through the society of skeletal radiology membership.

Information collected regarding MRI type and format includes the presence or absence of contrast administration, sequence types performed, and in which planes they were performed. These factors were not included in the MRI scoring report but were used to assess the appropriateness of imaging workup and to test for confounding between experimental groups. For each MRI report, the presence or absence of the following key elements was recorded: clinical history (as provided by the referring provider); relationship of the mass to vital and vascular structures; bone involvement (cortical violation, osseous infiltration, or smooth scalloping/remodeling); peritumoral edema; internal heterogeneity; tumor depth; evidence of necrosis; tumor borders or infiltration into adjacent tissues; malignant lymphadenopathy; use of multimodal imaging; tumor vascularity; recommendations for additional studies; description of the mass as anomalous; provision of a diagnostic impression; documentation of suspected malignancy; recommendation for biopsy and histopathological analysis; referral to a specialized sarcoma center; and correlation of imaging findings with the final diagnosis. Quality of history provided was not evaluated. Diagnostic correlation was defined as radiologic recognition of sarcoma (i.e., naming or clearly favoring sarcoma/malignant soft-tissue tumor). Agreement with the exact histopathologic subtype was not required.

These MRI-assessment items were developed collaboratively by two fellowship-trained musculoskeletal radiologists and a senior orthopedic oncologist at our institution. Several elements were adapted from a prior MRI quality-assessment study as well as guidelines established by the European Society of Musculoskeletal Radiology (ESSR), including those that describe the lesion and others that help guide clinical management [9]. Each descriptive item was scored dichotomously (present = 1, absent = 0) with equal weight. The report quality score was expressed as the percentage of applicable items documented: (items present ÷ applicable items) × 100. Lesion-specific items were not included in the MRI quality score.

## 3. Statistical Analysis

Descriptive statistics were used to summarize the study variables. Continuous variables were reported as means with standard deviations, while categorical variables were summarized as frequencies and percentages. To explore associations between categorical variables, chi-square tests for independence were performed, including Monte Carlo corrections when appropriate. Fisher’s exact tests were applied for comparisons with small cell counts. Correlations between continuous or ordinal variables were examined using Spearman’s rank correlation coefficients.

All statistical analyses were conducted using R (version 4.3.3) or an equivalent statistical software package. A significance level of 0.05 was used for all hypothesis tests.

## 4. Results

### 4.1. Radiologist Fellowship Training

A total of 100 MRI reports were analyzed, with 61 performed by general radiologists and 39 by musculoskeletal (MSK) fellowship–trained radiologists. There were 86 unique reading radiologists. Table 1 demonstrates the breakdown of STS subtype. Average size of the largest diameter for sarcomas that were reported was 8.9 cm; however, 11 reports failed to mention size at all. Several MRI reporting characteristics showed statistically significant differences between the two groups. Reports by MSK radiologists more frequently documented juxtaposition to vital structures (97.4% vs. 82.0%, *p* = 0.029), size (100% vs. 82%, *p* ≤ 0.0001), bone involvement (89.7% vs. 68.9%, *p* = 0.029), peritumoral edema (61.5% vs. 39.3%, *p* = 0.050), juxtaposition to vascular structures (66.7% vs. 55.7%, *p* = 0.028), and mention of malignancy (76.9% vs. 62.3%, *p* = 0.019). MSK radiologists were also more likely to recommend referral to a sarcoma center (30.8% vs. 3.3%, *p* = 0.001). “Malignant lymphadenopathy” was identified more often in reports by general radiologists (13.1% vs. 0%, *p* = 0.028) (Table 2).

### 4.2. Referring Provider Specialty

Among the 100 cases, 49 were referred by orthopedic providers and 51 by non-orthopedic providers. There were 95 unique reporting providers. Reports from orthopedic referrals were more likely to include patient history (89.8% vs. 68.6%, *p* = 0.022) and document juxtaposition to vital structures (98.0% vs. 78.4%, *p* = 0.007). No other MRI characteristics showed significant differences between referral groups.

### 4.3. MRI Report Scoring

Using the 19-point MRI quality score, MSK radiologists achieved higher average scores compared to general radiologists (62.3 vs. 54.7, *p* < 0.05), as seen in Figure 1. No significant differences in scores were observed between orthopedic and non-orthopedic referrals (60.3 vs. 56.7, *p* > 0.05). The distribution of scores is shown in Table 3, with average score comparisons by group in Table 4 and illustrated in Figure 2.

### 4.4. Correlation Analyses

Among general radiologists, significant positive correlations for variable reporting were observed between depth and bone involvement (r = 0.47, *p* < 0.001), depth and juxtaposition (r = 0.52, *p* < 0.001), and juxtaposition and border infiltration (r = 0.43, *p* < 0.001). Among MSK radiologists, significant correlations included depth and bone involvement (r = 0.50, *p* < 0.001), border infiltration and vascularity (r = 0.44, *p* = 0.011), gadolinium administration and heterogeneity (r = 0.44, *p* = 0.001), and mass detection and mention of malignancy (r = 0.42, *p* = 0.001) (Table 5).

### 4.5. Diagnostic Correlation

Overall, MRI report findings correlated with final histopathologic diagnosis in 45 of 100 cases (45%). Reports authored by MSK radiologists showed higher rates of diagnostic correlation compared to general radiologists (60.0% vs. 40.0%, *p* = 0.027). Several MRI characteristics were associated with improved diagnostic correlation, including documentation of heterogeneity (66.7% vs. 33.3%, *p* = 0.003), tumor depth (95.6% vs. 76.4%, *p* = 0.010), gadolinium administration (68.9% vs. 45.5%, *p* = 0.030), detection of a soft-tissue mass (100% vs. 89.1%, *p* = 0.031), drawing a diagnostic conclusion (55.6% vs. 29.1%, *p* = 0.013), reporting suspected malignancy (97.8% vs. 43.6%, *p* < 0.001), and necrosis (37.8% vs. 20.0%, *p* = 0.041). No associations were found for provider specialty, bone involvement, border infiltration, malignant lymphadenopathy, or multimodal imaging correlation. On multivariate analysis, heterogeneity, GAD administration, and MSK radiology fellowship training remained statistically significant. Depth trended towards significance, while necrosis was not statistically significant.

## 5. Discussion

This study evaluated the quality of initial MRI reports for patients with suspected soft-tissue sarcoma prior to assessment at a specialized sarcoma center. Overall, documentation of key MRI characteristics was limited, with most features reported in fewer than 70% of cases. Juxtaposition to vital structures was the most consistently documented element (88%), whereas other critical findings—such as peritumoral edema, bone involvement, and tumor vascularity—were noted far less frequently. Reports authored by musculoskeletal (MSK) fellowship–trained radiologists documented key characteristics more comprehensively, achieved higher overall MRI quality scores, and more often recommended referral to a sarcoma center compared with those by general radiologists. Several individual MRI features, including heterogeneity, depth, gadolinium use, and necrosis, were significantly associated with improved diagnostic correlation. Deficiencies in the quality of initial MRI reports for patients with suspected STS are well documented. In a series of 126 patients, Weiss et al. found that 67% of reports lacked one or more required sequences or planes, and 32% misclassified malignant masses as benign [9]. Korthaus et al. similarly reported persistent deviations from ESSR guidelines, even after guideline dissemination [16]. The updated ESSR consensus further emphasizes that reports should specify the exact anatomical location and include fluid-sensitive fat-saturated and T1-weighted sequences in at least two planes, with contrast administration when appropriate [17]. Omission of these elements can alter the diagnostic pathway. For example, Mankin et al. reported that inappropriate biopsy techniques lead to poorer outcomes and complicate definitive surgical management [18]. Alamanda et al. likewise noted that inadequate initial workup and unplanned excisions increase the risk of recurrence and metastasis [19]. These issues have direct clinical consequences, particularly for patients first imaged in nonspecialist settings, where early recognition of sarcoma is often missed and referral to specialized centers is delayed. Prompt referral to specialized centers for this rare disease has been repeatedly emphasized. Alvarez et al. showed that care at a cancer center was associated with significantly improved survival in adolescents and young adults, while Frassica et al. demonstrated decades earlier that specialized referral is critical for optimizing outcomes [20,21].

Prior studies have also shown that specific MRI features strongly correlate with identification of malignancy. Zhao et al. identified peritumoral enhancement as the strongest independent predictor of high-grade sarcoma (OR 13.6), while Scalas et al. reported that size, depth, heterogeneity, and necrosis correlate with worse outcomes [22,23] This convergence is reproducible across independent cohorts: Crombé et al. isolated peritumoral enhancement, necrosis, and heterogeneous T2 signal as the features associated with grade III disease and with worse metastasis-free and overall survival, and a systematic review by Schmitz and Sedaghat confirmed size, necrosis, peritumoral edema, and peritumoral enhancement as the descriptors most reproducibly linked to FNCLCC grade, while cautioning that definitions remain heterogeneous [24,25]. Fisher et al. reviewed functional MRI applications such as DWI and DCE, concluding that features like necrosis and peritumoral edema remain highly relevant for grading and prognosis [26]. The present analysis is consistent with these findings, specifically seen in Table 5 and Table 6, particularly regarding heterogeneity, depth, and necrosis, which were significantly associated with diagnostic correlation. More recent approaches have integrated intra-tumoral and peritumoral characteristics into predictive models, underscoring the continued importance of features that are often omitted in routine reports [27]. It is important to note that the design of this study cannot estimate specificity for malignancy. Our findings speak to sensitivity of recognition and to report completeness/quality, not to discriminating benign from malignant lesions.

Several limitations should be acknowledged. The retrospective design employed in this investigation introduces inherent biases, including reliance on the completeness and quality of existing radiology reports without access to underlying images for independent re-evaluation. The sample size, while comparable to prior studies, may limit the generalizability of subgroup analyses, particularly for comparisons between orthopedic and non-orthopedic referrals. Variability in MRI equipment, protocols, and institutional resources across sites was not controlled for and may have influenced the frequency with which certain features were documented. Additionally, the classification of radiologists as MSK trained using publicly available data may serve as a limitation and introduce some bias. Further, imaging conspicuity and likelihood of correct recognition differ by subtype and size, which was not evaluated in this study. Quality of initial history was not evaluated. Additionally, radiologists may not comment on very obvious findings. Additionally, there were 86 unique reading radiologists and 95 unique referring providers, which indicates that the observations may not be statistically independent and the results should be reviewed with caution. Strengths of the study include systematic evaluation of 19 ESSR-recommended MRI features, comparison of radiologist fellowship training and referral specialty, and correlation of individual MRI features with histopathologic diagnosis, providing a comprehensive assessment of reporting quality.

This study expands on the prior literature by not only quantifying the frequency of key MRI features but also demonstrating their association with diagnostic correlation, which earlier quality assessments largely did not address [9,16]. Evidence was provided that subspecialty training meaningfully impacts reporting quality, as MSK radiologists more consistently documented essential features and achieved higher diagnostic concordance. Furthermore, the clinical consequences of insufficient reporting are well documented. Inadequate description of tumor characteristics has been linked to inappropriate biopsy, unplanned excisions, higher risk of residual disease, and poorer outcomes [18,19,28,29]. Referral to specialized sarcoma centers has consistently improved survival and local control [20,21]. By directly linking underreporting of critical MRI features to diagnostic correlation, this study highlights a potential pathway through which suboptimal MRI reports contribute to delays, inappropriate interventions, and adverse outcomes.

In summary, initial MRI reports for STS frequently lack documentation of essential imaging characteristics. This is especially true of reports generated by non-MSK radiologists, whereas MSK fellowship–trained radiologists provide higher-quality reports with greater diagnostic concordance. These findings align with prior investigations but also extend them by demonstrating the link between specific MRI features and diagnostic correlation. The novelty of this study lies in its comprehensive evaluation of individual report elements and its sub-analysis of radiologist training and referral specialty. Future work should focus on developing structured reporting systems or scoring frameworks for STS MRI to standardize inclusion of critical features and improve diagnostic reliability. With advances in MRI technology and radiomics, such frameworks have the potential to ultimately improve patient care and would benefit from direct evaluation in future studies. The socioeconomic implications of insufficient imaging, including increased costs due to repeated procedures or advanced-stage disease at presentation, represent an additional area for future investigation.

## 6. Conclusions

This study demonstrates that initial MRI reports for soft-tissue sarcomas frequently omit essential diagnostic features and that subspecialty training in musculoskeletal radiology is associated with more complete reporting and improved diagnostic concordance. Implementation of structured reporting frameworks incorporating the key features identified here, particularly heterogeneity, depth, necrosis, and gadolinium enhancement, may help standardize STS evaluation across practice settings and reduce downstream diagnostic errors.

## Figures and Tables

**Figure 1:**
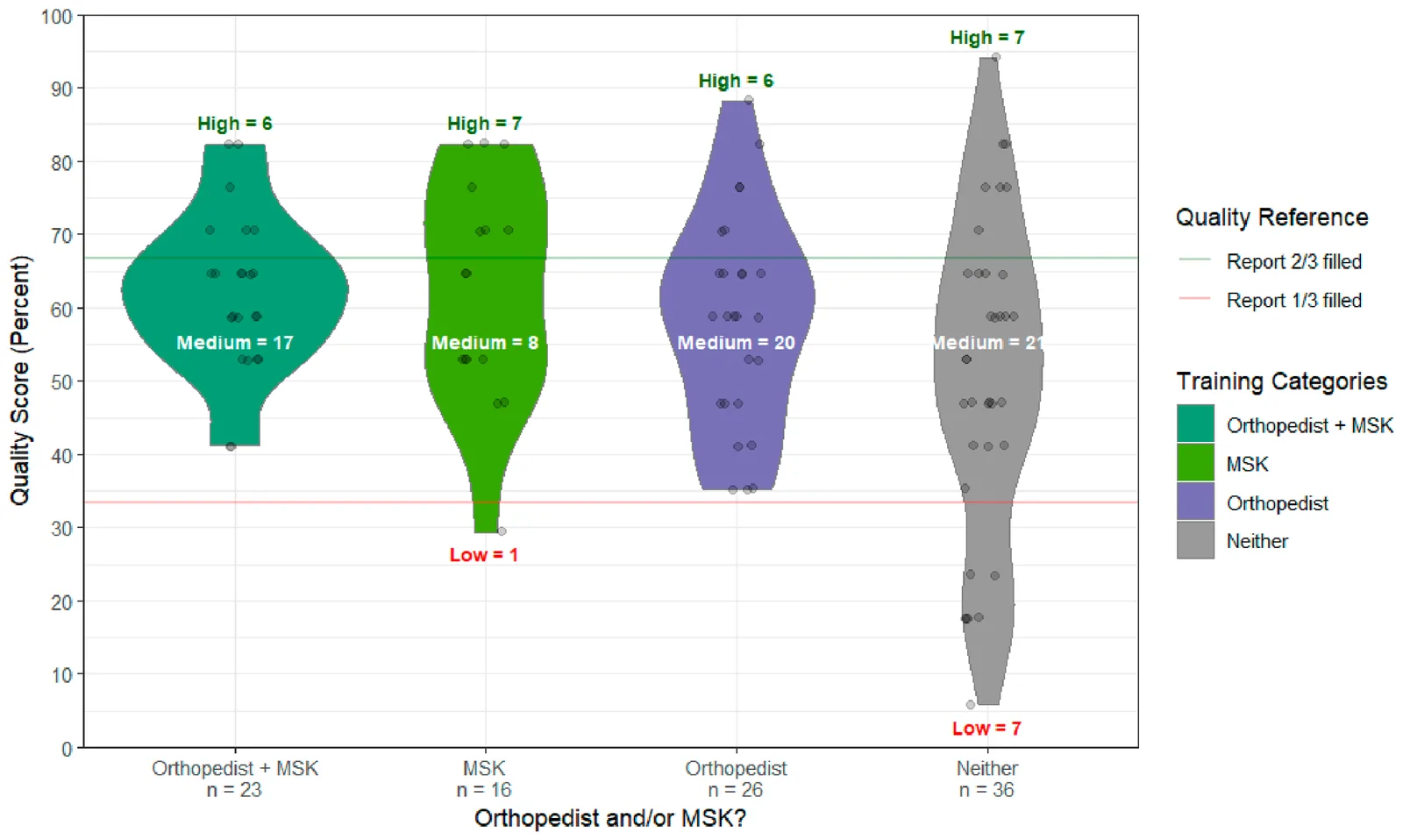
Violin plots combine boxplot-like statistics (e.g., median, spread) with a smoothed density shape, offering a dual insight into both the summary and the full distribution of scores. Each dot represents a quality score for an individual MRI report and they are stratified by Orthopedist and radiologist fellowship training.

**Figure 2:**
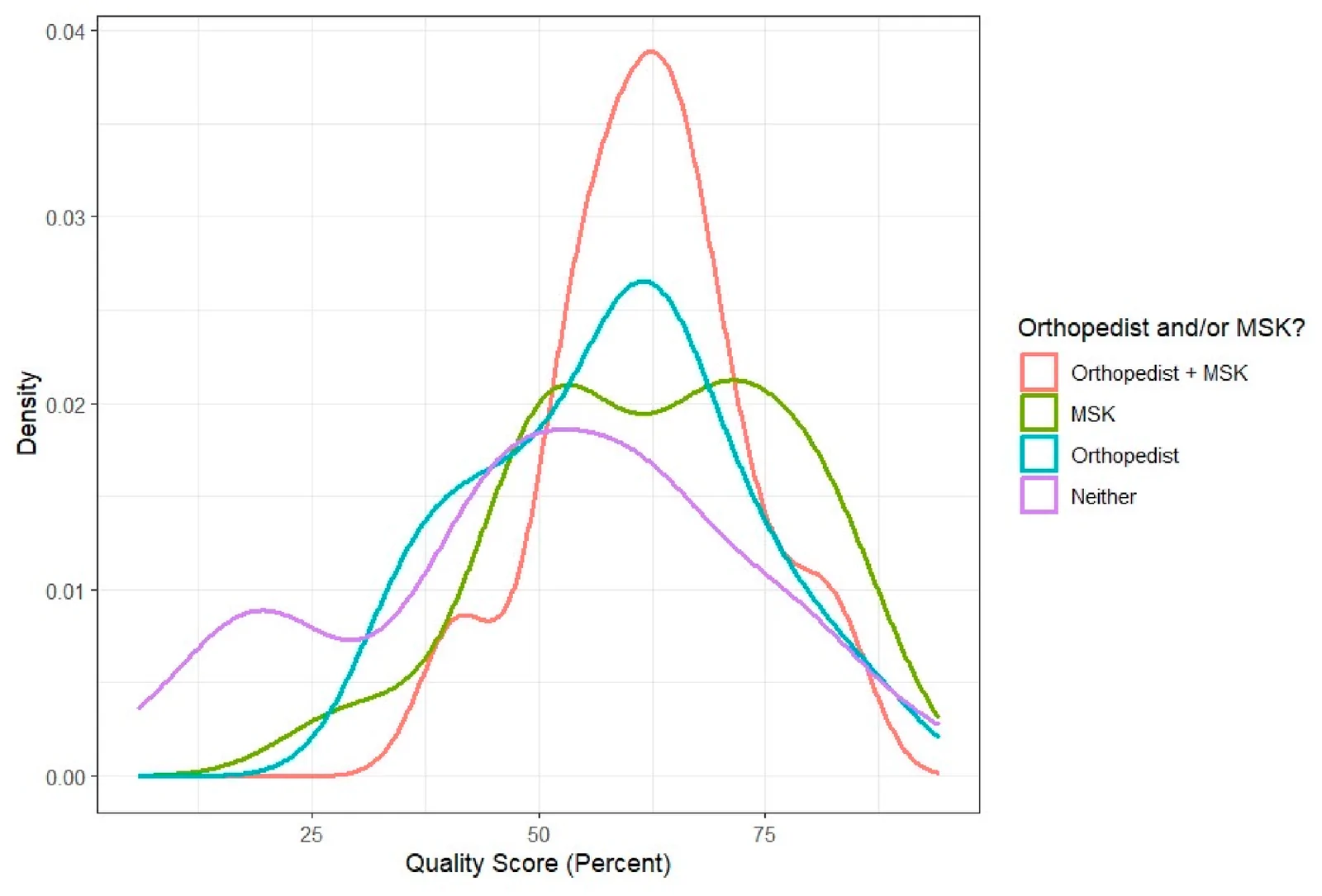
MRI score distribution. Density distribution of quality scores stratified by radiology fellowship training and referring provider type.

**Table 1:** Soft-tissue sarcoma count breakdown.

Sarcoma Type	N
Dedifferrentiated liposarcoma	1
Epithelioid sarcoma	1
Extraskeletal myxoid chontrosarcoma	2
Fibroblastic/myofibroblastic sarcoma	15
Leiomyosarcoma	11
Liposarcoma	9
Malignant peripheral nerve sheath tumor	2
Myofibrosarcoma	17
Myxoinflammatory fibroblastic sarcoma	3
Pleiomorphic cell sarcoma	1
Pleiomorphic fibroblastic sarcoma	1
Pleiomorphic leiomyosarcoma	1
Pleiomorphic myofibroblastic sarcoma	3
Pleiomorphic sarcoma	4
Pleomorphic liposarcoma	1
rhabdomyosarcoma	8
Round cell sarcoma	1
Solitary fibrous tumor	1
Spindle cell rhabdomyosarcoma	1
Spindle cell sarcoma	1
Synovial sarcoma	9
Undifferentiated pleiomorphic sarcoma	7
Total	100

**Table 2:** Presence/absence of the mention of specific characteristics of an initial MRI report and their association with radiologist fellowship training.

Characteristic	Generalist		MSK		Total		*p*-Value
	Patients	%	Patients	%	Patients	%	
History provided							
0	13	21.31	3	7.69	16	16	0.1259
1	48	78.69	36	92.31	84	84	
Juxtaposition to vital structures							
0	11	18.03	1	2.56	12	12	0.029
1	50	81.97	38	97.44	88	88	
Size							
0	11	18.00	0	0.00	11	11	<0.0001
1	50	82.00	39	100.00	89	0	
Bone involvement							
0	19	31.15	4	10.26	23	23	0.0294
1	42	68.85	35	89.74	77	77	
Peritumoral edema							
0	37	60.66	15	38.46	52	52	0.0498
1	24	39.34	24	61.54	48	48	
Heterogeneity							
0	32	52.46	19	48.72	51	51	0.8729
1	29	47.54	20	51.28	49	49	
Depth							
0	12	19.67	3	7.69	15	15	0.1772
1	49	80.33	36	92.31	85	85	
Necrosis							
0	46	75.41	26	66.67	72	72	0.4706
1	15	24.59	13	33.33	28	28	
Border infiltration							
0	33	54.1	17	43.59	50	50	0.4122
1	28	45.9	22	56.41	50	50	
Malignant lymphadenopathy							
0	53	86.89	39	100	92	92	0.0275
1	8	13.11	0	0	8	8	
Vascularity of mass							
0	47	77.05	28	71.79	75	75	0.7225
1	14	22.95	11	28.21	25	25	
Juxtaposition to a vascular structure							
0	27	44.26	13	33.33	40	40	0.028
1	34	55.74	26	66.67	60	60	
Completely imaged							
0	1	1.64	0	0	1	1	0.9999
1	60	98.36	39	100	99	99	
GAD administered							
0	23	37.7	16	41.03	39	39	0.903
1	38	62.3	23	58.97	61	61	
Other studies recommended							
0	40	65.57	16	41.03	56	56	0.0274
1	21	34.43	23	58.97	44	44	
Mass detected							
0	4	6.56	2	5.13	6	6	0.9999
1	57	93.44	37	94.87	94	94	
Conclusion drawn							
0	36	59.02	23	58.97	59	59	0.9999
1	25	40.98	16	41.03	41	41	
Report mentions malignancy							
0	23	37.7	9	23.08	32	32	0.0190
1	38	62.3	30	76.92	68	68	
Referral to sarcoma center							
0	59	96.83	27	69.23	86	86	0.001
1	2	3.27	12	30.77	14	14	
Multimodal imaging correlation							
0	46	75.41	29	74.36	75	75	0.9999
1	15	24.59	10	25.64	25	25	

**Table 3:** Scoring of initial MRI reports based on 19 key characteristics and their association with radiologist fellowship training. A Score of 17, 18, and 19 was not included due to there being a total score of 0.

Score	Generalists	MSK	Total	% Generalists	% MSK
1	1	0	1	1.64	0.00
2	0	0	0	0.00	0.00
3	4	0	4	6.56	0.00
4	2	0	2	3.28	0.00
5	0	1	1	0.00	2.56
6	4	0	4	6.56	0.00
7	5	2	7	8.20	5.13
8	9	2	11	14.75	5.13
9	4	8	12	6.56	20.51
10	10	5	15	16.39	12.82
11	9	8	17	14.75	20.51
12	3	6	9	4.92	15.38
13	5	2	7	8.20	5.13
14	3	5	8	4.92	12.82
15	1	0	1	1.64	0.00
16	1	0	1	1.64	0.00
Total	61	39	100	61.0	39.0

**Table 4:** Average score association with radiologist fellowship training and referring provider.

Radiologist Training Type	Average	n	Standard Error (SE)	Group
Generalist	54.65	61	2.18	A
MSK	62.32	39	2.74	B

**Table 5:** Presence/absence of the mention of specific characteristics of an initial MRI report and their association with diagnosis correlation.

Characteristic	Total n = 100 Mean (Std)/n (%)		Diagnosis Correlated n = 45 Mean (Std)/n (%)		Diagnosis Not Correlated n = 55 Mean (Std)/n (%)		*p*-Value
**MSK or General**							**0.027**
General	61	61.0	18	40.0	43	78.2	
MSK	39	39.0	27	60.0	12	21.8	
History provided							1.000
No	16	16.0	7	15.6	9	16.4	
Yes	84	84.0	38	84.4	46	83.6	
Juxtaposition to vital structures							**0.050**
No	12	12.0	2	4.4	10	18.2	
Yes	88	88.0	43	95.6	45	81.8	
Bone involvement							0.685
No	23	23.0	9	20.0	14	25.5	
Yes	77	77.0	36	80.0	41	74.5	
Presence or absence of peritumoral edema							0.243
No	52	52.0	20	44.4	32	58.2	
Yes	48	48.0	25	55.6	23	41.8	
**Heterogeneity**							**0.003**
No	51	51.0	15	33.3	36	65.5	
Yes	49	49.0	30	66.7	19	34.5	
**Depth**							**0.010**
No	15	15.0	2	4.4	13	23.6	
Yes	85	85.0	43	95.6	42	76.4	
Lesion completely imaged							0.450
No	1	1.0	1	2.2	0	0.0	
Yes	99	99.0	44	97.8	55	100.0	
**GAD administered**							**0.03**
No	44	44.0	14	31.1	30	54.5	
Yes	56	56.0	31	68.9	25	45.5	
Other studies recommended							0.903
No	56	56.0	26	57.8	30	54.5	
Yes	44	44.0	19	42.2	25	45.5	
**Necrosis**							**0.041**
No	72	72.0	28	62.2	44	80.0	
Yes	28	28.0	17	37.8	11	20.0	
Borders/infiltration							0.108
No	50	50.0	18	40.0	32	58.2	
Yes	50	50.0	27	60.0	23	41.8	
Malignant lymphadenopathy							0.727
No	92	92.0	42	93.3	50	90.9	
Yes	8	8.0	3	6.7	5	9.1	
Multimodal imaging correlation							0.296
No	75	75.0	31	68.9	44	80.0	
Yes	25	25.0	14	31.1	11	20.0	
Vascularity							0.131
No	75	75.0	30	66.7	45	81.8	
Yes	25	25.0	15	33.3	10	18.2	
Juxtaposition to vascular structures?							0.838
No	40	40.0	17	37.8	23	41.8	
Yes	60	60.0	28	62.2	32	58.2	

**Table 6:** Multivariate logistic regression: predictors of diagnostic correlation.

Variable	Adjusted OR	95% CI	*p*-Value
Heterogeneity	3.05	1.21–7.66	0.018
Depth	4.63	0.92–23.31	0.063
Necrosis	1.69	0.63–4.53	0.302
GAD administered	3.19	1.71–5.83	0.029
MSK radiologist	2.86	1.46–4.89	0.047

## Data Availability

Data can be made available by the authors upon reasonable request.
